# Sugar and Dyslipidemia: A Double-Hit, Perfect Storm

**DOI:** 10.3390/jcm12175660

**Published:** 2023-08-31

**Authors:** Alejandro Gugliucci

**Affiliations:** Glycation, Oxidation and Disease Laboratory, Touro University California, Vallejo, CA 94592, USA; alejandro.gugliucci@gmail.com

**Keywords:** fructose, glucose, insulin resistance, liver fat, TRL, ANGPTL, apoCIII, chylomicrons, VLDL, LDL, LPL, atherogenesis, remnants, apoB100, apoB48

## Abstract

The availability of sugar has expanded over the past 50 years, due to improved industrial processes and corn subsidies, particularly in the form of sweetened beverages. This correlates with a surge in the prevalence of cardiometabolic disorders, which has brought this issue back into the spotlight for public health. In this narrative review, we focus on the role of fructose in the genesis of cardiometabolic dyslipidemia (an increase in serum triglyceride-rich lipoproteins (TRL): VLDL, chylomicrons (CM), and their remnants) bringing together the most recent data on humans, which demonstrates the crucial interaction between glucose and fructose, increasing the synthesis while decreasing the catabolism of these particles in a synergistic downward spiral. After reviewing TRL metabolism, we discuss the fundamental principles governing the metabolism of fructose in the intestine and liver and the effects of dysregulated fructolysis, in conjunction with the activation of carbohydrate-responsive element-binding protein (ChREBP) by glucose and the resulting crosstalk. The first byproduct of fructose catabolism, fructose-1-P, is highlighted for its function as a signaling molecule that promotes fat synthesis. We emphasize the role of fructose/glucose interaction in the liver, which enhances de novo lipogenesis, triglyceride (TG) synthesis, and VLDL production. In addition, we draw attention to current research that demonstrates how fructose affects the activity of lipoprotein lipase by increasing the concentration of inhibitors such as apolipoprotein CIII (apoCIII) and angiopoietin-like protein 3 (ANGPTL3), which reduce the catabolism of VLDL and chylomicrons and cause the building up of their atherogenic remnants. The end outcome is a dual, synergistic, and harmful action that encourages atherogenesis. Thus, considering the growing concerns regarding the connection between sugar consumption and cardiometabolic disease, current research strongly supports the actions of public health organizations aimed at reducing sugar intake, including dietary guidance addressing “safe” limits for sugar consumption.

## 1. Introduction

Obesity, diabetes, and related cardiometabolic disorders, such as metabolic-associated fatty liver disease (MAFLD)—also called non-alcoholic fatty liver disease (NAFLD)—are on the rise [1,2,3,4,5,6]. These epidemics have a complex etiology that is influenced by interactions between heredity and environmental factors, including nutrition and physical exercise. The availability of sugar has expanded over the past 50 years, particularly in the form of sweetened beverages, due to improved industrial processes and corn subsidies [7,8,9,10]. This is associated with a surge in the prevalence of cardiometabolic disorders. Sugar is added to beverages and food products in the form of sucrose or high-fructose corn syrup (HFCS). Both are rich sources of glucose and fructose and have a fructose content of at least 50%. The most popular formulation added to beverages is called “HFCS 55” and contains 45% glucose and 55% fructose [1,11]. Diets high in fructose can promote hypertriglyceridemia in both animals and people much more rapidly than diets containing comparable amounts of starch or glucose, a finding that sparked interest in fructose as a cause of metabolic disease in the 1960s. Hypertriglyceridemia caused by sugar and carbohydrates was closely linked to hyperinsulinemia in both obese humans and animals [1,2,3,5,6,12,13,14,15,16,17,18,19,20,21,22].

Numerous articles bear witness to the significance of fructose as a lipogenic substrate that increases liver fat and causes hypertriglyceridemia [10,13,19,22,23,24,25,26,27,28,29,30,31]. Nevertheless, some authors contend that the issue of whether added sugars and naturally occurring dietary sugar contribute to the epidemic of cardiometabolic disease is still up for debate, arguing that the issue ultimately comes down to calories and obesity [32,33,34,35]. However, a closer look at the funding sources of most of the studies making these assertions reveals that they originate from the food/beverage industry and are biased in that direction. These publications intend to stall the advancement of science by creating uncertainties, in a manner comparable and practically identical to that used by the cigarette industry decades ago [36,37,38]. Another, albeit well-intended, source of controversy is the use of an experimental design regarding overfeeding fructose alone, which introduces another kind of bias since humans consume fructose mainly as either sugar or HFCS. Before being absorbed, sucrose must be broken down into its monosaccharide components, glucose and fructose, by intestinal microvilli disaccharidase, while HFCS contains the free monosaccharides.

Therefore, this narrative review highlights the fact that an important nutritional cause of dyslipidemia, which, in turn, affects the risk of cardiovascular disease (CVD), is the concurrent, daily, and chronic flux of coupled fructose and glucose (as sugar or HFCS) to the liver. Accordingly, we concentrate on the specific roles that fructose plays in the development of this cardiometabolic dyslipidemia (an increase in triglyceride (TG)-rich lipoproteins—TRL—comprising VLDL, chylomicrons, and their remnants). We bring together the most recent data, which show how glucose and fructose interact critically to simultaneously increase the synthesis and decrease the catabolism of these particles in a synergistic downward spiral. After discussing the fundamental principles governing the metabolism of TRL (by the liver, enterocyte, and vascular surfaces), we outline the mechanisms for the increased production of TRL. Briefly, the current review summarizes fructose/glucose pathways in those organs, focusing on the effects of dysregulated fructolysis and on the resulting crosstalk that promotes fructolysis. We then underscore the role of fructose/glucose interaction in the liver, which enhances de novo lipogenesis, TG synthesis, and VLDL production. Regarding the other side of the coin, the delayed catabolism of TRL, we draw attention to current research that demonstrates how fructose hampers TRL intravascular catabolism by affecting the activity of lipoprotein lipase (LPL) through a rise in the concentration of inhibitors such as apoCIII and ANGPTL3. This reduces the catabolism of VLDL and CM, causing the buildup of their atherogenic remnants. We are consequently in the presence of a dual, synergistic, and harmful action of sugar that encourages atherogenesis. Hence, we hope that this review will reveal that current research strongly supports the actions of public health organizations aimed at reducing sugar intake, including dietary advice addressing “safe” limits for sugar consumption.

## 2. Sugar in Excess Produces Unwanted Metabolic Consequences

### 2.1. Sugar in Food, and More So in a Liquid Form, Is Hazardous; Metabolic Consequences Are Far Worse When It Is Provided in Liquid Form and on an Empty Stomach

Obviously, the direst consequences occur when the intake is large, fast, and chronic [4,5,13,20,39,40,41]. The World Health Organization advises that adults and children should consume less than 10% (ideally less than 5%) of their energy needs from free sugar to lower their risk of developing obesity and metabolic disorders [10,15,41]. This roughly corresponds to 330 mL of soda or fruit juice. The consumption of 25% of energy requirements in the form of sugar is very frequent in the USA, especially in children and adolescents [13,31]. According to the US Department of Agriculture and the US Department of Health and Human Services, sugar-sweetened beverages (SSBs) are the main source of added sugar and fructose intake in both children and adults, and consumption of these beverages is consistently linked to an increased risk of cardiometabolic disease, including the elevated cardiovascular mortality linked to poor diet [21,30,39,42,43,44]. In addition to the mono- and disaccharides that are naturally present in honey, syrups, fruit juices, and fruit juice concentrates, free sugars also include the mono- and disaccharides that are added to foods and beverages. Recent investigations on the use of sugar across Europe, Latin America, and the USA discovered that the mean sugar intakes in most of these regions were greater than the advised amount. As a result, many nations are discussing or have already implemented steps to minimize sugar intake, such as improved food labeling or tariffs on foods that are sweetened [45,46,47].

### 2.2. “Healthy and Natural” Fruit Juice Is Also a Culprit

Notwithstanding the above, not all the blame should be ascribed to “added sugars”. The main solid ingredient in fruit juices is also free sugar, which can range in concentration from 100 to 120 g/L, depending on the type and quality of the fruit [20,41,48,49]. Most natural fruit juices have a fructose concentration that is comparable to HFCS-55-sweetened beverages. For instance, orange juice typically contains 51–57 g/L of total fructose (including free fructose and fructose from sucrose), or 52–54% of its total sugar content. A glass of orange juice comes from 4–5 oranges. Usually, people do not eat that number of oranges in a sitting; moreover, when eating the whole fruit, a large amount of fiber is ingested, which retards the absorption of the free sugars [20]. Apple juice poses another issue: the overall amount of sugar is similar but the ratio of fructose to glucose is about 2. This poses a second problem. Fructose-to-glucose ratios of greater than 1:1 are referred to as excess free fructose (EFF). A glass of 100% apple juice has 8 to 9 g of EFF. After a 25 and 12 g EFF challenge, respectively, 30% and 10% of participants in a study tested positive for fructose malabsorption; these intake levels are easily met by people who consume large amounts of apple juice [50,51,52,53]. As shall be examined later, excess free fructose has deleterious consequences on the microbiota and intestinal health that add to the impact of fructose on its main target, the liver.

## 3. Triglyceride-Rich Lipoproteins (TRL): Energy-Rich Healthy Particles in Healthy Quantities, Villains When in Excess

In the last ten years, a wealth of knowledge regarding the function of TRL metabolism and the ensuing remnant particles in atherogenesis has become available [26,27,54,55,56,57,58,59]. This knowledge has also stimulated the discovery of new therapeutic targets. Prior to focusing on how sugar affects their metabolism, it is useful to review the primary pathways of TRL metabolism, highlighting the recently discovered role of some apolipoproteins, the crucial physiological role of lipoprotein lipase and its main regulators, the significance of these particles’ fluxes in the postprandial period, their catabolic rate, and the partition of TRL during the feed-fast cycle.

### 3.1. Production of Circulating TRL

TG offer a reliable and portable energy source. For a substantial portion of their evolutionary history as hunter-gatherers, humans had relatively occasional access to food containing high amounts of fat, necessitating the development of metabolic systems to store and maintain available TG for utilization in an orderly way. Highly controlled and effective processes are used to transfer TG between the sites of absorption (intestine), storage (adipose tissue), repackaging (liver), and utilization (muscle) to make the most of this resource. Circulating TRL originate from the liver and are carried by TG, generated by the body through an endogenous TRL pathway. TG derived from meals are converted by the intestines into chylomicrons. Both routes contain parallels and dissimilarities that we will describe in the following sections.

#### 3.1.1. Endogenous Routes: The Liver Produces VLDL throughout the Day, Which Is Crucial during Fasting

VLDL carries TG, which is made in the liver, to the body’s peripheral tissues, where they are used. While fasting, these lipoproteins carry more than 90% of the TG in the blood. Overall, over 50 g of fat is delivered daily in this manner. These TG are derived from a variety of fatty acid (FA) sources, such as chylomicron remnants and hepatic de novo lipogenesis (DNL), which is the process by which carbohydrates are converted into FA, as well as the uptake of non-esterified (free) fatty acids (FFA) from the plasma and the release of FA from hepatocyte cytosolic lipid droplets [26,27,55,57].

**Figure 1:**
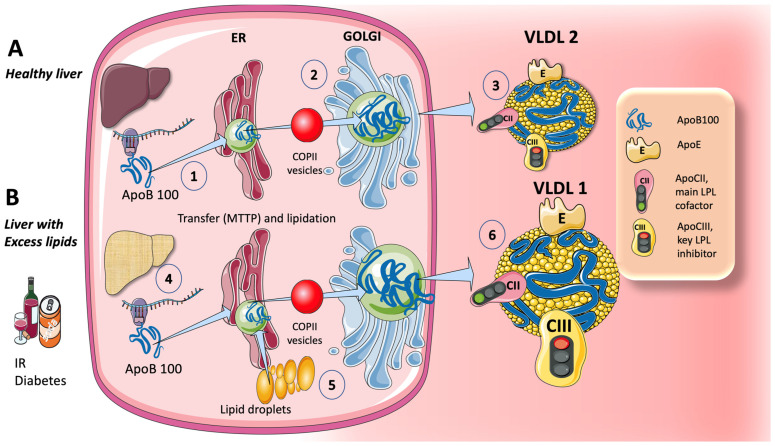
Production of endogenous triglyceride-rich lipoproteins (TRL) by the liver. (**A**) Healthy liver. (1) ApoB100 is synthesized by hepatocytes in the reticulum (ER) where it is moved along by the concourse of microsomal triglyceride transfer protein (MTTP). (2) Lipidated apoB100 transfers to the Golgi apparatus by means of COP II vesicles. (3) Finally, very low-density lipoprotein 2 (VLDL2), containing phospholipids and cholesterol besides triglycerides, is secreted into the circulation. (**B**). Liver with excess lipids. (4) Triglycerides employed for the synthesis of VLDL may come from re-esterified fatty acids catabolism of remnants or de novo lipogenesis (DNL). DNL implies the synthesis of fatty acids from acetyl COA derived from the metabolism of glucose and fructose. DNL is only 1–5% in normal livers, but it reaches 25% when there is insulin resistance, diabetes, and the high consumption of sugars or alcohol. Excess fat from DNL, build-up of circulating fatty acids, or liver steatosis (5) is employed to enrich VLDL2 into a much larger particle called VLDL1 (6). As compared to VLDL2, VLDL1 is not only a larger particle but also contains more apoCIII. As we illustrate later, apoCIII is a very potent inhibitor of the catabolism of VLDL, thereby promoting hypertriglyceridemia. This figure was partly generated using Servier Medical Art, provided by Servier, licensed under a Creative Commons Attribution 3.0 unported license.

We propose a simplified figure (Figure 1) to illustrate the complex and tightly controlled assembly process for VLDL. The initial stage in this process, which results in the formation of a pre-VLDL particle, is the co-translational lipidation of the growing apoB100 polypeptide in the endoplasmic reticulum (ER), which is mediated by the microsomal TG transfer protein (MTTP). When the chaperones separate and the lipidation is adequate to allow the correct folding of apoB100, pre-VLDL can be further lipidated to produce VLDL2, a lipoprotein with a small, triglyceride-rich core (Figure 1A). One of the biggest proteins known, apoB100 has a mass of 500 kDa. Larger, triglyceride-rich VLDL1 is produced normally, but considerably more is produced in livers with extra fat (1 B), by merging the developing VLDL2 with lipid droplets [26,27,57].

The synthesis of VLDL1 increases when hepatic TG levels are high, probably to protect hepatocytes from the negative consequences of TG overload (Figure 1B). Insulin, a crucial metabolic regulator of energy storage and consumption (namely, fat and carbohydrate metabolism), has a major impact on VLDL1 production. Both directly and indirectly (by lowering FFA flow into the liver), insulin prevents the release of VLDL1 from the liver [27,60,61]. Postprandially, when insulin levels are high, the direct suppression of VLDL1 synthesis occurs. VLDL2 is, in a nutshell, the smaller, “healthier” version of VLDL.

Both the direct and indirect actions of hormones are compromised in persons with type 2 diabetes mellitus, high levels of liver fat, and insulin resistance [5,60,62,63,64]. Alcohol and sugar both cause a dose-dependent increase in hepatic FA synthesis and a dose-dependent decrease in FA oxidation, which has the overall impact of boosting VLDL secretion (as we will explore in more detail later in this article).

Other than apoB100, as seen in Figure 1, VLDL1 and VLDL2 include a variety of surface apolipoproteins, many of which are absorbed from HDL in the circulation. The most significant ones, which will be considered in our discussion, are apoCII, the primary activator of lipoprotein lipase (key to the release of FA from TG in TRL, to be taken up by tissues), and apoCIII, the primary inhibitor [27,65].

#### 3.1.2. Exogenous Routes: In the Postprandial Period, Enterocytes Produce Chylomicrons

**Figure 2:**
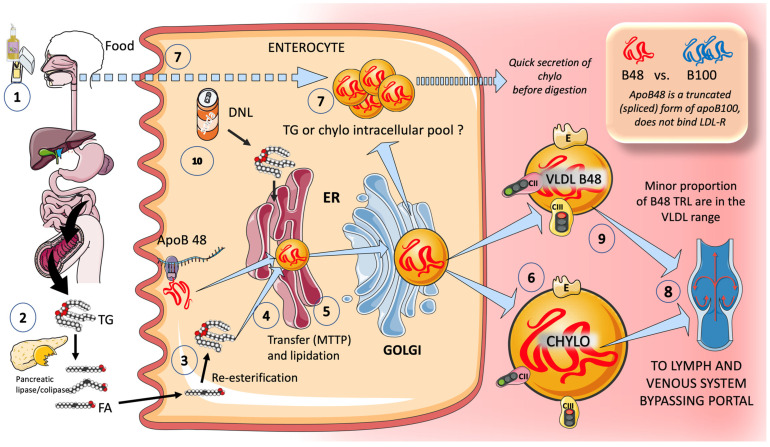
Production of exogenous triglyceride-rich lipoproteins (TRL) by the intestine via chylomicrons. (1) Lipids in the diet are (2) digested by pancreatic lipase, which is activated by liver co-lipase, resulting in the absorption of fatty acids and monoglycerides. (3) Fatty acids are re-esterified by the enterocyte; the resulting triglycerides (4) serve to lipidate apoB48. The latter is a truncated, spliced form of apoB100 that lacks the LDL receptor binding sites. (5) The resultant chylomicrons, which are very large particles, are (6) secreted into the circulation. (7) However, very recently, it has been shown that some of them are either stored as an intracellular pool or are swiftly produced from TG droplets that can be called upon very quickly when the next meal comes, following signals coming from the intake of more food even before it reaches the intestines. (8) Chylomicrons secreted into the circulation contain phospholipids, cholesterol, and liposoluble vitamins, and are first transported by the lymph, then reaching the venous circulation and finally the arterial circulation in a third step. It has recently been shown that some of the apoB48-containing lipoproteins coming from the intestines are also in the VLDL size range (9). Sugar provides two lipogenic substrates (10). This figure was partly generated using Servier Medical Art, provided by Servier, licensed under a Creative Commons Attribution 3.0 unported license.

We present a simplified diagram of the key elements of enterocyte chylomicron formation in Figure 2. Except for short-chain fatty acids, all dietary fat (more than 70 g per day) is transported by the chylomicron assembly pathway [66,67,68]. Many hypotheses have been proposed to explain the formation of these massive TRL. As depicted in the Figure, pancreatic lipase breaks down dietary fat with the help of liver co-lipase in a complex system of micelles that ultimately results in the generation of free fatty acids and monoglycerides. After being absorbed by the enterocyte, these fatty acids are re-esterified into TG. This serves as a first point of control over the relative proportions of saturated versus mono, poly, unsaturated fatty acids in these molecules. ApoB48, the protein that makes up the core of chylomicrons, is a truncated, spliced version of apoB100 that lacks the ability to bind to the LDL receptor. As in the case of VLDL, but through a less well-known process, apoB48 is lipidated with TG, phospholipids, and cholesterol as it travels through the ER and Golgi apparatus. Primordial, lipid-poor, apoB48-containing lipoproteins and large CM may assemble through different processes. CM that have only just been secreted, as seen in Figure 2, are released into the lymphatic vessels and travel to the thoracic duct, where they are delivered to the left subclavian vein along with their cargo of dietary lipids and fat-soluble vitamins. As a result, unlike other nutrients, most dietary lipids do not penetrate the hepatic portal system [69,70]. As we shall see, a fructose overload may also increase the formation of CM and DNL in the intestine [66,67,70,71,72].

### 3.2. Catabolism of TRL

**Figure 3:**
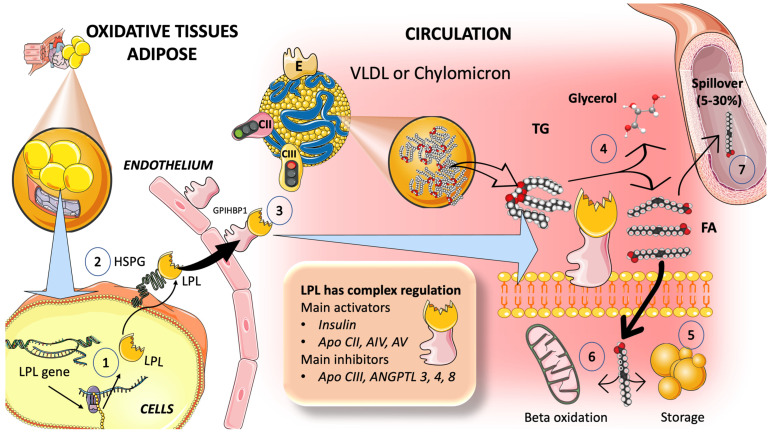
Lipoprotein lipase is the main regulator of TRL fluxes. (1) Lipoprotein lipase is synthesized by the cells in oxidative tissues, such as muscle-skeletal and myocardial tissues, as well as in adipocytes and mammary glands, among others. (2) LPL is an extremely complex enzyme that requires heparin sulfate proteoglycans (HSPG) on the surface of the cells to help in the transcytosis of the molecule to the luminal face of the capillaries. (3) Glycosylphosphatidylinositol-anchored high-density lipoprotein binding protein 1 (GPIHBP1) serves to anchor LPL on the luminal surface of the endothelial cells and aids in the conformation of LPL to an active lipolytic enzyme. (4) LPL acts on TRL to hydrolyze triglycerides into glycerol and free fatty acids. Free fatty acids are employed for storage in adipocytes, as shown in (5), or for oxidation in muscle and myocardial tissue, as shown in (6). (7) Some of the fatty acids (which may be from 5 to 30%) remain in the circulation and as referred to as spillover fatty acids. LPL has a very complex regulation, its main activators being insulin, apoCIII, AIV, and AV, and its main inhibitors being apoCIII and angiopoietin-like protein (ANGPTL) 3, 4, and 8. The argument that the delayed turnover or catabolism of TRL is more significant than excess production in the pathophysiology of hypertriglyceridemia is supported by the most recent findings. This figure was partly generated using Servier Medical Art, provided by Servier, licensed under a Creative Commons Attribution 3.0 unported license.

Strict control over TG transport is necessary for survival. Over the past ten years, significant regulatory components have been discovered that help us fully understand TRL metabolism. Lipoprotein lipase (LPL), which catalyzes the release of FA from TG in TRL for storage by adipose tissue or for use as an energy source by muscle, is the main enzyme in the intravascular breakdown of TG in circulating TRL (Figure 3). LPL is not produced by the liver, but rather by macrophages, adipose tissue, skeletal and cardiac muscle, and the brain. LPL operates at the endothelium luminal surface in those tissues [73,74].

#### 3.2.1. Multiple Modulators Work Together in the Intricate Regulation of LPL Synthesis and Activity

For instance, insulin appears to control LPL activity in adipocytes at both the post-transcriptional and post-translational levels (Figure 3). The rapid adaptive adjustments of LPL to dietary changes are governed by insulin responses, together with the regulating effects of other hormones and proteins that control necessary variations in the lipolytic rates between fasting and postprandial states (Figure 3). Numerous apolipoproteins present on the surface of TRLs have various effects on their metabolism. ApoCI, apoCII, apoCIII, and apoE are exchanged between TRL and HDL particles, but not apoB, depending on the nutritional or metabolic state [73,74,75,76,77,78,79,80]. Studies show that LPL activity is increased by apoCII and apoAV and decreased by apoCI, apoCIII, and apoE.

#### 3.2.2. TRL and HDL Dynamic Intravascular Crosstalk

Concurrent with the entry of chylomicron and VLDL particles into plasma from the intestine and liver, respectively, exchangeable apolipoproteins (apos) from HDL are acquired [81,82,83,84,85]. These include apoCII, which causes TG hydrolysis to begin by activating lipoprotein lipase, apoAV, which promotes this process, along with others, most notably apoCIII and apoCI, which can impair it. Additional proteins that can be derived from HDL include apoE, apoAI, and apoAI. Following the onset of lipolysis, as depicted in Figure 3, some exchangeable apos are shed to HDL, and phospholipid and cholesteryl ester transfer proteins (CETP), respectively, mediate the exchange of surface and core lipids with HDL and LDL [81,82,83,84,85].

Since CETP transfers TG to HDL and cholesterol to the remnant or LDL particle, this process explains why hypertriglyceridemia and low HDL cholesterol levels are frequently associated [84,85,86]. As a result, low HDL cholesterol is not harmful in and of itself, but is rather a sign of TRL dyslipidemia. It serves as a useful surrogate marker of the presence of atherogenic TRL remnants in the circulation [23,81,82,87]. The importance of HDL in these processes cannot be overemphasized. Nevertheless, details are outside the purview of this review.

#### 3.2.3. TRL Remnants Are at Least as Atherogenic as LDL

Together, the activity of LPL and the transfer of apos, TG, and CE result in particles known as remnants: chylomicron (apoB48) and VLDL (apoB100) remnants, respectively.

Remnant particles are hydrolyzed further by hepatic TG lipase (HL), during passage through the hepatic sinusoids, and gain additional apoE; this facilitates their binding and uptake by proteins on the surface of the liver cells, such as the LDL receptor (LDLR), LDL-like receptor protein-1 (LRP-1), and the heparin sulfate proteoglycan syndecan-1, for final disposal [55,56,87,88,89,90]. Plasma chylomicron metabolic products typically remain relatively large, lipid-rich, and buoyant, but as the VLDL remnants are gradually broken down, IDL particles are created, which then advance to LDL. We will expand on these topics along with the latest research on the deleterious role of sugar in LPL regulation. To efficiently incorporate both processes, let us first discuss sugar metabolism.

## 4. Sugar Metabolism: A Bird’s Eye View on the Axis of Enterocyte-Liver-Systemic Organs

### 4.1. Fructose Has a Very Low Serum Concentration; the Liver Acts as a Very Effective First-Pass Buffer

The pronounced discrepancies in their circulating blood levels reflect the intrinsic distinctions between mammalian glucose versus fructose metabolism. Fructose circulates at less than 0.02 mM under fasted settings, compared to the normal fasting blood glucose concentration of 5 mM. For the past 50 years, conventional thinking has held that the liver is primarily responsible for the removal of fructose [45,91,92,93]. However, recent research produced a fresh and significant idea that intestinal fructose metabolism, at low intake doses, might protect the liver from excessive exposure to dietary fructose, where its metabolism may be particularly harmful. The significance of intestinal versus hepatic fructose metabolism in larger animals, including humans, must be further clarified by in-depth research [91,92,93].

### 4.2. Enteral and Hepatic Fructose Metabolism

**Figure 4:**
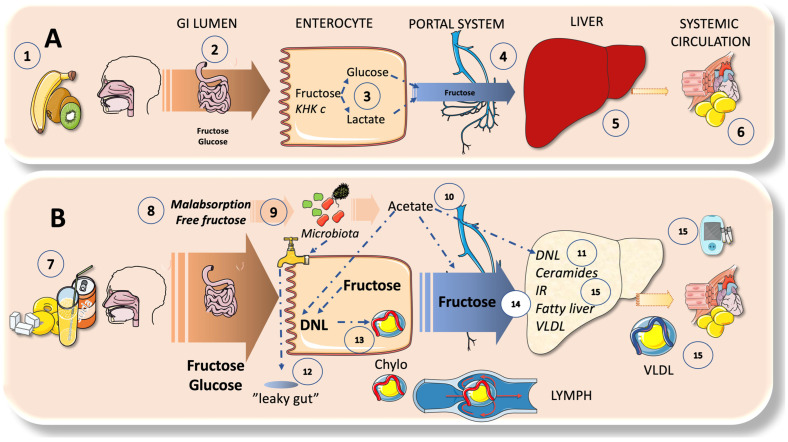
Outline of the enteral versus hepatic metabolism of sugar: the differential effect of small versus large intakes. (**A**). When the ingestion is of small doses of sugar, such as in fruit (1), the intestines (2) act as a barrier and can metabolize fructose to glucose or lactate (3), which is then circulated via the portal system (4) to the liver (5) and next to the systemic circulation (6). (**B**). When the consumption is much higher (7), such as that of a large proportion of American teenagers (which can amount to up to 25% of the caloric intake), several deleterious reactions occur: malabsorption of fructose (8) is frequent because glucose is absorbed against a concentration gradient in an active way, whereas fructose needs a concentration gradient; i.e., glucose absorption is faster. Residual fructose feeds the microbiota (9), which (among other effects) produce acetate that is then directed to the liver via the portal system (10) and participates in fat synthesis by de novo lipogenesis (DNL) (11). Fructose promotes the growth of unwanted microbiota, which, in turn, are responsible for leaky gut (12), which allows unwanted molecules to reach the liver with deleterious inflammatory effects. Recent data in humans show that DNL occurs in the intestines and contributes to and increases the secretion of chylomicrons (13). A saturated enteral barrier allows for the escape of much of the absorbed fructose into the portal system (14), inundating the liver and producing an array of deleterious reactions (15) that result in either fatty liver or dyslipidemia, as discussed further in this review. Note: KHK—ketohexokinase. This figure was partly generated using Servier Medical Art, provided by Servier, licensed under a Creative Commons Attribution 3.0 unported license.

Figure 4 succinctly contrasts intestine and liver fructose metabolism under two different dietary intakes. Utilizing an energy-independent route, fructose is absorbed through the apical membrane of intestinal epithelial cells. This process calls for the transmembrane transporter protein, GLUT5. In response to the availability of fructose, GLUT5 expression and function are elevated, increasing the absorption of dietary fructose. The enzyme fructokinase is required for the intracellular metabolism of fructose in the cytosol in this process. The related GLUT2 transporter, which also aids in the basolateral transport of absorbed glucose and galactose, is responsible for the bulk of absorbed fructose entering the circulation over the basolateral membrane of enterocytes [45,47,91,92]. When modest amounts of sugar are consumed, as in fruit, the intestines act as a barrier and can convert fructose to glucose or lactate, which is subsequently transported to the liver via the portal system. When consumption is much higher, as it is in many American teenagers (where it might make up to 25% of calorie intake), various negative effects can occur. The remaining fructose nourishes the microbiota; this, among other activities, allows them to release acetate, which is then transported to the liver via the portal system and takes part in the process of DNL, which produces FA [91,94,95,96]. Fructose fosters the development of undesirable microbiota, which, in turn, causes leaky gut; this enables detrimental substances to enter the liver and causes detrimental inflammatory consequences. Studies on rodents show that changes in the gut microbial composition, including increases in *Sutterella, Coprococcus*, and other bacteria, paired with a decrease in the abundance of Firmicutes bacteria, are associated with the negative effects of high-fructose feeding, such as MetS and oxidative stress. The ability of dual probiotics to reduce fructose-induced MetS has also been demonstrated [91,94,97].

### 4.3. DNL Can Also Occur in the Intestines

Recent research in humans demonstrates that DNL also takes place in the intestines and increases the release of chylomicrons [4,18,20,45]. Much of the absorbed fructose can escape through a saturated enteral barrier and enter the portal system, where it overwhelms the liver and causes a variety of harmful reactions that either cause fatty liver or dyslipidemia, as is further addressed in this review.

## 5. Sugar and Increased TRL Production and the Key Role of the Liver

### 5.1. A Set of Three Unique Enzymes Are Used in the Non-Tightly Controlled, Primarily Hepatic, Metabolism of Fructose

**Figure 5:**
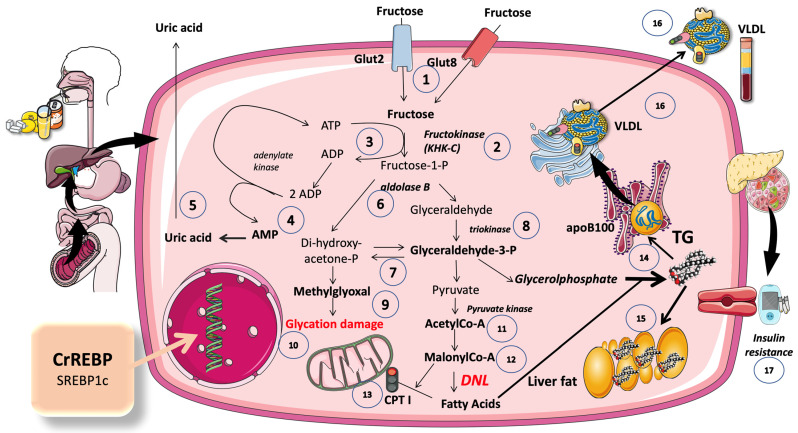
Main pathways of fructose metabolism by the liver. For the sake of clarity, only fructose reactions are shown in this figure, on the understanding that they occur at the same time as glycolysis. The importance of the interactions between glucose and fructose metabolism in the liver is explained in the text, as well as in Figure 3 and Figure 4. More than 90% of absorbed fructose is retained in the liver. Fructose from the portal vein enters the hepatocyte (1) via transporter Glut 2 (a high-capacity transporter also used by glucose) and, secondarily, by Glut 8. The phosphorylation of fructose requires a specific enzyme, either fructokinase or ketohexoquinase C (KHK C) (2). This phosphorylation is very quick and is unregulated (unlike glucose phosphorylation by glucokinase) and may lead to the depletion of ATP if the concentration of fructose is very high (3). Ultimately, the recycling of ADP leads to the production of AMP (4), which is then transformed into uric acid (5). Another enzyme that is specific for the metabolism of fructose is aldolase B (6), which leads to the production of the classic trioses of glycolysis (7) with the aid of triokinase (8). An increase in the flux of trioses may lead, under certain circumstances, to the production of methylglyoxal (9) and advanced glycation end products (10) that damage proteins, lipids, and DNA. Since, usually, fructose is ingested at the same time and in the same amounts as glucose, which is converted largely into glycogen or sent to the circulation, the extra trioses are mostly shunted to acetyl-coA (11) and then enter the pathway for the synthesis of fatty acids, also known as de novo lipogenesis (DNL), via malonyl-coA (12). This molecule commits these carbons to fat as it inhibits CPT1 (13), hindering FA entry into the mitochondria for oxidation. Either by providing the carbons as shown in the figure, or by stimulus (ChREBP and SREBP1c), as discussed in the text and in Figure 3 and Figure 4, fructose provides the trioses for the backbone of triglycerides (TG) and the fatty acids as well (14). TG may accumulate as liver fat (15), or be secreted as VLDL (16) or both, depending on fluxes, other substrates, genetics, and hormones. Overactive DNL, caused by a constant excess of fructose, also leads to the production of ceramides, which may be the basis of hepatic insulin resistance, further complicating the metabolic derangement (17). This figure was partly generated using Servier Medical Art, provided by Servier, licensed under a Creative Commons Attribution 3.0 unported license.

The liver metabolism of fructose accounts for more than 80% of the total load. Three enzymes work in a cascade to break down fructose as soon as it enters the cell. In a nutshell, as illustrated in Figure 5, fructose is effectively converted by these fructolytic enzymes into triose-phosphates, which are then added to the cellular pools of triose-phosphates produced by glycolysis and gluconeogenesis [12,16,18,20,45]. Depending on fluxes, hormone environment, and other factors, different portions of the trioses pool turn into acetyl-coA, glycerol backbones in TG, glycogen, lactate, or fatty acids in the process called DNL. Ketohexokinase c (KHKc), commonly known as fructokinase, catalyzes the quick and irreversible phosphorylation of fructose into fructose-1-phosphate (Fructose-1-P), which is the initial step in fructolysis. This metabolite does not participate in glycolysis or gluconeogenesis and is unique to the fructolytic pathway. Fructose is a weak substrate for hexokinases when compared to glucose. KHKc is not allosterically inhibited by ATP, nor by other signaling molecules of sufficient cellular energy, its immediate product. In the human liver, the ability of KHKc to phosphorylate fructose is ten times greater than the ability of glucokinase (GK) to phosphorylate glucose. Furthermore, while hepatic GK is sequestered in the nucleus in an inhibited state by the glucokinase regulatory protein (GKRP), KHKc is constitutively active [45]. Therefore, most of the fructose that enters the liver via the portal circulation is rapidly removed and little reaches the systemic circulation, due to the low Km (very high affinity), high activity, and lack of control of KHKc regarding fructose [45,46].

### 5.2. An Ingenious Evolutionary Defense System Gone Awry

From an evolutionary perspective, this is clearly a good mechanism to assimilate a rare, sporadic commodity in the diet (fructose) as quickly and efficiently as possible. Discernibly, this mechanism, in our current time, is a liability. In hepatocytes, Fructose-1-P can quickly build up to millimolar concentrations. As a result, if intake is high and rapid (SSB; fruit juices), the fructose phosphorylation-induced decrease in free phosphate effectively activates AMP deaminase and uric acid synthesis, as depicted in Figure 5 [98,99,100]. Uric acid has a key role in hypertension, promotes further fructose metabolism, and impairs mitochondrial function [43,101]. As was already mentioned, the gut is capable of significant fructose metabolism, and KHKc is expressed in both the liver and the intestine. The findings are consistent with the theory that intestinal fructose metabolism may protect the liver from fructose intake, where its metabolism may be very damaging. Another KHK isozyme, KHKa, is widely expressed at low levels.

### 5.3. Excess Trioses Not Only Feed TG Production but also Produce Toxic Metabolites

As shown in Figure 5, fructose contributes to the synthesis of methylglyoxal molecule (MG), a potent precursor of the advanced glycation end products (AGE). Trioses generated from the uncontrolled metabolism of fructose may boost the liver’s ability to produce MG [20,102]. This increase in MG causes dicarbonyl stress, which is characterized by protein and DNA modifications and malfunctions (MG targets the arginine residues in particular). We have previously proposed that the enzyme adenosyl-monophosphate-dependent kinase (AMPK), which, in a healthy liver, would activate the catabolic pathways, is inactivated by MG [103]. When coupled to arginine residues in critical AMPK functional pockets, MG. may inactivate AMPK, thereby favoring the anabolic processes, including lipogenesis and IR, which are widely related to obesity, metabolic syndrome, and NAFLD. D-lactate is produced during the detoxification of MG. Trioses, MG, and D-Lactate excess can increase when large amounts of fructose are consumed. Since the plasma levels of D-lactate have been utilized as a stand-in indicator of MG flow, this substance is of special interest. Elevated MG and D-lactate levels have been shown in obese adults and we have demonstrated the same in teenagers in a cross-sectional research project [104,105]. Furthermore, in our fructose restriction study, a 38% reduction in D-lactate levels occurred in just 10 days [22].

### 5.4. DNL Is Greatly Enhanced When Glucose and Fructose Are Metabolized at the Same Time (Sugar or HFCS, Its Usual Source)

As was previously mentioned, the possible negative effects of fructose on metabolism were first identified because of its detrimental effects on lipid homeostasis. This includes the ability of very high fructose exposure to induce fasting hypertriglyceridemia and steatosis and to increase postprandial lipemia after only a few of days of exposure. Increased hepatic DNL, the metabolic mechanism by which new fatty acids are generated from precursor molecules, is consistently linked to these alterations in the liver and circulating TG levels. Accordingly, let us briefly introduce the subject here.

#### 5.4.1. DNL Transforms Extra Dietary Carbohydrates (CHO) into Fatty Acids (FA)

As shown in Figure 5, FA are produced during this process from acetyl-CoA molecules generated directly from CHO catabolism (such as glycolysis or fructolysis), or from the acetate produced by fructose fermentation by the microbiota [106]. DNL requires the expression of enzymes involved in the lipogenic pathway by a variety of cell types, especially white adipocytes and hepatocytes. ATP-citrate lyase (ACLY), acetyl-CoA carboxylase (ACC), and fatty acid synthase (FASN) are three important enzymes that facilitate glucose or fructose-derived DNL [16,31,106,107]. Citrate, from acetyl-coA in the mitochondrial Krebs cycle, acts as a carbon shuttle to the cytosol. Citrate in the cytosol is next broken down by ACLY into acetyl-CoA and oxaloacetate. The 2-carbon building block for future lipogenesis is this cytosolic acetyl-CoA. Acetyl-CoA is then transformed into malonyl-CoA by ACC, particularly the cytosolic isoform, ACC1. Malonyl-CoA units are subsequently put together by FASN in a multi-step process to create the 16-carbon palmitate, which is the first lipogenic end product [15,16,22,31]. Elongases and desaturases work together to convert palmitate into various lipid species. Importantly, the same malonyl-CoA prevents FA entry into the mitochondria, shifting the substrate to fat synthesis and not oxidation [1,13,48,108,109,110]. Systemic insulin sensitivity must be maintained by a proper balance of hepatocyte and adipocyte DNL. The master transcription factors (discussed below) that control the expression of lipogenic enzymes are the carbohydrate-responsive element-binding protein (ChREBP), which is driven by CHO intake, and sterol-responsive element-binding protein-1 (SREBP-1), which is induced by CHO intake/insulin signaling.

DNL supplies FA for the preservation of the cell’s structural integrity, permits the storage of CHO energy outside of the glycogen store (contributing to glucose homeostasis), and modulates FA oxidation. It has previously been determined that the liver’s FA production mechanism is particularly relevant to understanding the causes of dyslipidemia and MAFLD; the latter is beyond the scope of this review. Studies that examined the impact of higher CHO/sugar/fructose consumption show that even when maintenance dietary interventions are implemented, the consumption of carbohydrates, especially when they are ingested as simple sugars and in liquid form, promotes hepatic lipogenesis [1,13,17,24,48,108,109,111,112]. Fructose is a more effective inducer of hepatic lipogenesis than glucose, according to research using fructose and glucose treatments. There is proof that sugar/fructose intake affects hepatic DNL from both intervention trials that raised sugar/fructose intake and those that decreased fructose intake [13,15,17,19,21,22,40,41,44,49,66,113,114]. Importantly, the few studies that specifically examined the effects of other hexoses (such as glucose and fructose) provide evidence in favor of the idea that fructose is a more effective inducer of lipogenesis than glucose. It is important to bear in mind that DNL is a source of new FA, but the liver synthesizes TG using other sources, FA from plasma, FA from remnants, and FA from storage droplets.

#### 5.4.2. Inhibition of KHC-c Blocks Fat Synthesis

The relative contribution of tissues to fructose metabolism depends on the levels of expression of the enzymes in the fructolytic pathway. KHK-C is regarded as a crucial enzyme in the metabolism of fructose because, as previously mentioned, it phosphorylates fructose at a rapid rate. KHK-C deletion and KHK-C blockage are both effective for protection against fructose-induced metabolic disturbances [45,47]. In addition, due to an increased supply of fructose to the liver, deletion of the KHK-A isoform aggravates fructose-induced metabolic syndrome. Finally, according to clinical research, NAFLD patients have higher levels of KHK-C expression in their livers; decreasing the level of KHK-C causes a decrease in liver fat in NAFLD patients. All the evidence supports the development of KHC-c inhibitors, currently in the pipeline for humans [45,47].

### 5.5. Cross-Talk between Fructose and Glucose Metabolism in the Liver: The Role of F-1-P as a Signaling Molecule of Abundance That Has Gone Awry in Current Dietary Habits

**Figure 6:**
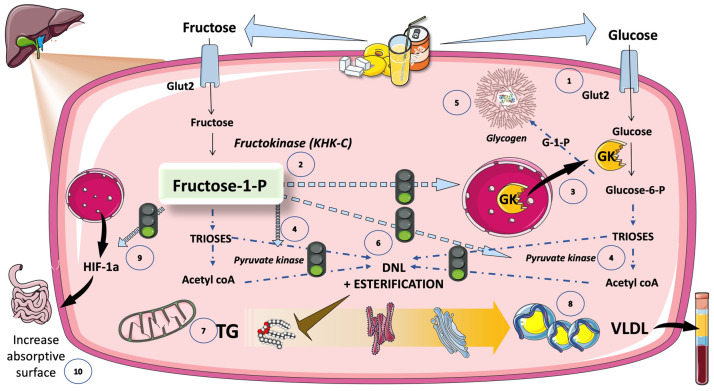
Crosstalk between fructose and glucose metabolism in the liver: the role of F-1-P as a signaling molecule of abundance that has gone awry in current dietary habits. The ingestion of fructose is usually accompanied by a similar amount of glucose, be it in the form of sugar or high-fructose corn syrup. Akin to fructose, glucose is also taken up by Glut 2 transporters in the hepatocytes (1). Fructose-1-P (2), the product of fructokinase, acts as a signaling molecule that activates glucose metabolism by its action on glucokinase, GK (3), and pyruvate kinase (4), resulting in an increased flux of intermediates. After the saturation of glycogenesis (5), these lead to DNL (6) and esterification, with TG as end products (7). TG are packed with apoB100 in VLDL (8) and/or stored as liver fat, as shown in Figure 2. Fructose-1-P induces the expression of hypoxia-inducible factor 1 subunit alpha (HIF1a) (9), which, in turn, promotes microvilli expansion and increased absorptive surface (10), thereby enhancing energy uptake and TG production. This figure was partly generated using Servier Medical Art, provided by Servier, licensed under a Creative Commons Attribution 3.0 unported license.

Fructose may be a more harmful sugar than glucose because of differences in the way each one is metabolized. As depicted in Figure 5, fructose only needs one phosphorylation precursor to produce DNL. This reaction by fructokinase to create fructose-1-phosphate leads downstream to the three-carbon molecule components, DHAP and GA. While DHAP and GA metabolism are controlled, fructokinase activity is not. More importantly, the ingestion of fructose is usually accompanied by a similar amount of glucose, be it in the form of sugar or as HFCS. As illustrated in Figure 6, akin to fructose, glucose is also taken up by Glut 2 transporters in the hepatocytes. Fructose-1-P, the product of fructokinase, acts as a signaling molecule that activates glucose metabolism by its action on glucokinase, GK, and pyruvate kinase [45,115]. These activations result in an increased flux of intermediates, which, after the saturation of glycogenesis, lead to DNL and esterification, with glycerol resulting in TG as the end products. Fructose-1-P thus functions as a signaling molecule that stimulates DNL. Evidence from fructose-intolerant patients and animal models, where increased fructose-1-P but decreased fructolytic flux are related to greater steatosis, supports this conclusion [115]. TG are packed with apoB100 in VLDL and/or stored as liver fat, as shown in Figure 6. Furthermore, fructose-1-P induces the expression of hypoxia-inducible factor 1 subunit alpha (HIF1a), which, in turn, promotes microvilli expansion and an increased absorptive surface, thereby enhancing energy uptake and TG production [115].

Increased fructolytic flow may additionally provide substrate and reducing equivalents to support fatty acid synthesis, in addition to the previously mentioned pathway. Additionally, it has been proposed that DNL is promoted by uric acid produced because of fast fructose metabolism, as shown in Figure 5. Fructose in “catalytic” concentrations can significantly raise the hepatic glucose levels. Additionally, it has been demonstrated that Fructose-1-P inhibits glycogen phosphorylase and activates pyruvate kinase, the last step in glycolysis, as shown in Figure 6. Fructose’s effects on raising hepatic Fructose-1-P, which results in the dissociation of GK from its inhibitory binding partner, GKPR, represent the main mechanism by which fructose produces DNL in the liver [16,31,106,116,117]. Fructose-1-P, as a signaling molecule, probably evolved as a useful mechanism for processing a scarce, intermittent dietary component (fructose) as quickly and effectively as is feasible. Clearly, this activity is a disadvantage in today’s world.

### 5.6. DNL Is Further Regulated by Transcription Factors Induced by Glucose and Fructose (Sugar or HFCS, the Usual Sources)

#### 5.6.1. Hexose- and Triose-Phosphate Concentrations in the Hepatocyte Rise Because of Increasing Glycolytic Flow and Fructolytic Flow

**Figure 7:**
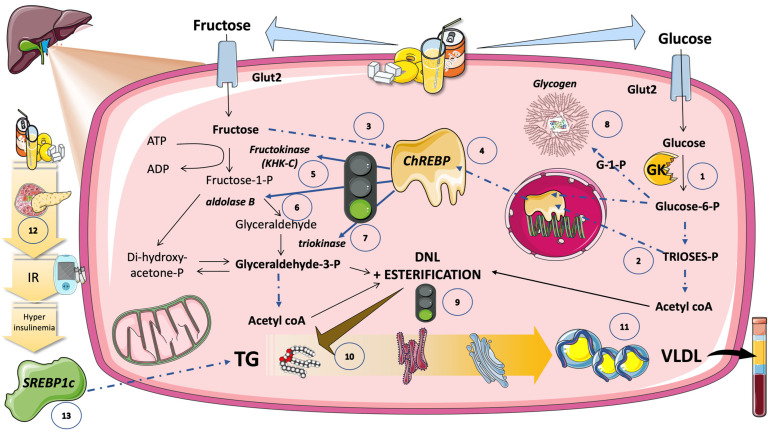
Crosstalk between fructose and glucose metabolism in the liver: the role of ChREBP as a transcription factor that has gone awry in current dietary habits. Glucose metabolites, mainly glucose-6-P (1) and trioses-P (2) and, possibly, fructose itself (3), induce the expression of ChREBP. ChREBP (4) is an evolutionarily conserved, carbohydrate-sensing transcription factor, necessary for the carbohydrate-mediated activation of glycolytic and lipogenic enzymes in the liver. The expression of the whole complement of fructolytic enzymes is also transactivated: fructokinase (5), aldolase B (6), and triokinase (7). After the glycogen stores are replenished (8), excess carbons are shunted to DNL and esterification (9), with TG as end products (10). TG are packed with apoB100 in VLDL (11) and/or stored as liver fat, as shown in Figure 2. Chronic hyperactivity of these pathways leads to hepatic IR (12) with the increased expression of SREBP1c (13), resulting in augmented lipid synthesis. ChREBP and SREBP1c have additive effects on targets that promote lipogenesis. Insulin dramatically stimulates SREBP1c activity. This figure was partly generated using Servier Medical Art, provided by Servier, licensed under a Creative Commons Attribution 3.0 unported license.

As illustrated in Figure 7, when one or more of these glucose metabolites (especially glucose-6-P) activate the carbohydrate-responsive element-binding protein (ChREBP), the entire set of enzymes needed for DNL is coordinately upregulated [118,119,120,121,122]. Enterocytes, hepatocytes, adipocytes, pancreatic beta cells, and proximal tubule cells of the kidney are just a few examples of the major metabolic cell types that express high levels of the evolutionarily conserved carbohydrate-sensing factor ChREBP. ChREBP is necessary for the carbohydrate-mediated activation of glycolytic and lipogenic enzymes in the liver. The expression of the whole complement of fructolytic enzymes is also transactivated [28,63,121,123,124,125]. The expression of hepatic ChREBP-beta, a powerful ChREBP isoform, as well as its fructolytic, glycolytic, and lipogenic targets, are immediately and robustly upregulated in vivo by the gavage of fructose but not glucose [121]. ChREBP liver-specific knockdown or deletion blocks the induction of DNL enzymes and DNL activity by sucrose and fructose.

#### 5.6.2. ChREBP Does Not Work Alone

Figure 7 further illustrates that ChREBP does not function in isolation to control the expression of DNL enzymes and other lipid homeostasis mediators. Another key regulator of lipogenic enzymes that reacts to nutrients and hormones is the sterol regulatory element-binding protein 1c (SREBP1c). ChREBP and SREBP1c have additive effects on targets that promote lipogenesis [126,127,128]. Insulin dramatically stimulates SREBP1c activity through a route that is reliant on AKT and mTORC1 (mechanistic target of rapamycin complex 1). Although fructose does not directly trigger the release of insulin, the increased adiposity and hyperinsulinemia brought on by chronic fructose ingestion may raise SREBP1c activity (Figure 7).

ChREBP and SPREBP1c, as well as other crucial metabolic transcription factors, including PPAR-g, PPAR-a, estrogen-related receptors, and the liver X receptor, can all have their transcriptional activity increased by the transcriptional coactivator PPAR-g coactivator 1b (PGC-1b). PGC-1b, thus, appears to support a variety of harmful sugar-mediated traits [126,127].

#### 5.6.3. Carbons vs. Stimulus

The role of fructose as a lipogenic substrate is sometimes challenged by industry-funded research using figures from studies that were conducted in the past using fructose in isolation [32,33,91,93]. These studies showed quantitative disposal pathways for fructose carbons, as follows: gluconeogenesis (28.9–54%), lactate synthesis (28%), and oxidation (30.5–62%), with just a small quantity of fructose being directed toward FA synthesis (1%). The limitations of these studies are obvious. First, as shown above, fructose intake is performed with a similar amount of glucose, yielding the consequences discussed above. Second, DNL is measured as carbons in FA that arise from fructose itself, thereby showing only a small part of the picture. Fructose does provide up to 30% of the glycerol backbone of TG and acts as a potent stimulus, via fructose-1-P, of DNL. Finally, when IR is present, even the carbons from fructose itself are found in FA on TG by up to 30% [26,27,60].

Fructose overfeeding and restriction studies show the involvement of fructose in the overproduction of TRL in humans [10,13,14,15,17,19,21,22,30,40,41,44,48,66,113,114,129,130,131,132]. Fructose effects on hepatic very-low-density lipoprotein (VLDL) metabolism, which influences the levels of total plasma TG, have been the subject of several dietary research studies in animals as well as humans. Recent interventional investigations, spanning weeks to months, have demonstrated that overfeeding humans with moderate to high doses of fructose-containing sugars can have a negative impact on their metabolic results. Over a two-week period, the addition of SSBs, ranging from 10% to 25% of the required energy, increased cardiovascular risk variables such as lipids and uric acid. In an important study, the authors evaluated the impact of giving persons who were overweight or obese isocaloric beverages that were sweetened with glucose or fructose for 10 weeks, accounting for 25% of their basal energy needs. Both treatments led to a similar increase in body weight. However, visceral adiposity, DNL, an atherogenic dyslipidemia, and the indicators of insulin resistance were all enhanced by fructose but not by glucose [15,40]. Restriction studies (including our own; see below), particularly those carried out in children and adolescents, provide the strongest clinical evidence that dietary sugar, when ingested in proportions that are typical of Western diets, correlates to unfavorable metabolic health. Dietary fructose intake significantly increases the risk of hepatic insulin resistance through the intricate interplay of numerous metabolic pathways, at least some of which are unrelated to increased weight gain and calorie intake overall. Additional research is required to fully comprehend the underlying mechanisms and to pinpoint areas where changed fructose metabolism can be employed to treat insulin resistance and fatty liver disease. Depending on the study population, the level of sugar restriction, and the length of the study, most studies demonstrated that interventions focused on reducing SSB consumption reduced weight gain, adiposity, liver fat, and the indicators of insulin resistance.

## 6. Sugar and Increased Intestinal TRL Production: Fructose May Be Involved in Increased Production of Chylomicrons

As argued earlier, TRL dyslipidemia occurs when TRL and their associated remnant particles are either produced in larger amounts or cleared more slowly (or both), which can lead to an increase in plasma TG concentration (which is a surrogate marker for TRL). In terms of dietary fructose metabolism, the liver is indeed thought to be the primary organ for this activity. However, the increased generation of chylomicron (CM) particles from the digestive tract may also play a role in fructose-induced dyslipidemia, according to some studies [66,67,133]. These effects have been demonstrated in both males with metabolic syndrome (MetS) and a hamster model of insulin resistance. Data from both in vivo and in vitro studies suggests that chylomicron remnants also contribute to the development of atherosclerotic plaques as do VLDL remnants [26,27,61,65,67,134]. The intestine may significantly contribute to plasma TG levels in participants with insulin resistance if a comparable link between the rates of hepatic and intestinal DNL occurs since hepatic DNL is enhanced several-fold in such participants. An increased risk of atherosclerosis is linked to elevated TRL, both when fasting and postprandial. However, even though postprandial lipids may be more informative, treatment guidelines are only determined by fasting lipid values.

### New Insights into Human Chylomicron Metabolism Have Been Provided by Recent Chylomicron Purification Techniques

Due to the challenges of ultracentrifugation (a large VLDL and small CM overlap), research into the functions of endogenous versus dietary fat has been hampered. VLDL contamination in chylomicron fractions is significant [31,66,71,113]. We and others have recently devised immunological methods that isolate pure chylomicrons [71,113]. The results demonstrated the effective separation of postprandial lipoproteins and substantially improved purity compared with ultracentrifugation protocols, by using the immunoaffinity method [19,57,71]. These methods can be used to better delineate the role of dietary sugar and fat on postprandial lipids in the context of cardiovascular risk and explore the potential role of fructose in increased intestinal DNL, CM remnants, and atherosclerosis. Exciting new information on these topics will, consequently, emerge throughout the coming years.

## 7. The Second Hit: Fructose and LPL, and the Role of the Main Inhibitors

The role of fructose in the production of TRL has been the main emphasis of research up to this point. In addition, recent evidence is accumulating to show that in the downstream arm, TRL hydrolysis is also compromised. Recent interventional investigations, spanning weeks to months, have demonstrated that overfeeding humans with moderate to high doses of fructose-containing sugars can have a negative impact on LPL regulation. These studies agree with our own restriction study. Together, they show that high fructose levels in the diet are associated with increased apoCIII, while fructose restriction reduces them [19,31,40,41,44,113].

### 7.1. Fructose and apoCIII

**Figure 8:**
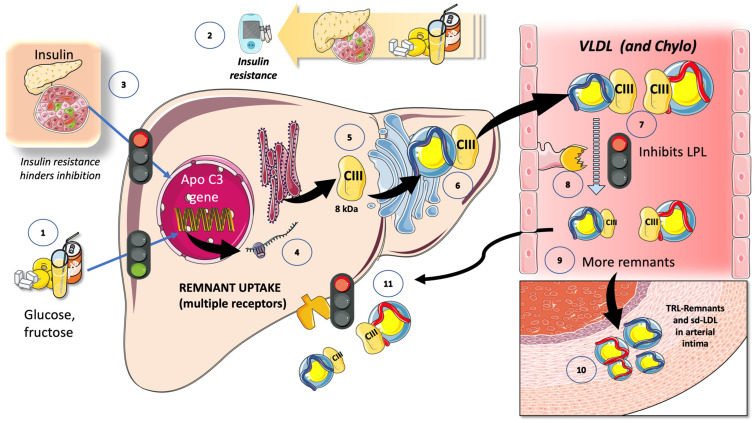
Fructose is associated with the increased expression of apoCIII, resulting in the impaired intravascular catabolism of VLDL and chylomicrons. Glucose (1), either directly, due to hyperglycemia, or indirectly (2) by producing insulin resistance (3), increases the liver’s expression of apoCIII (4,5). Glucose stimulates and insulin inhibits apoCIII production. Apo CIII in VLDL (6) and in chylomicrons (7) decrease the activity of LPL (8), delaying catabolism and thereby increasing remnant concentration (9). Remnants are atherogenic and some become small-dense LDL; they all accumulate in the arterial intima, promoting atherosclerosis (10). A minor (in humans) additive effect of apoCIII is to precisely impair the uptake of remnants by the liver (11). This figure was partly generated using Servier Medical Art, provided by Servier, licensed under a Creative Commons Attribution 3.0 unported license.

The important function of apoCIII as an LPL inhibitor and the findings of investigations on animal and human losses of function have heralded the potential relevance of apoCIII inhibitors as a treatment approach for hypertriglyceridemia. As we illustrate in Figure 8, sugar is associated with the increased expression of apoCIII, resulting in the impaired intravascular catabolism of VLDL and chylomicrons [19,26,134,135,136]. Glucose, either directly, due to hyperglycemia, or indirectly, by producing insulin resistance, increases the liver’s expression of apoCIII. Glucose stimulates and insulin inhibits apoCIII production. ApoCIII in VLDL and in CM decrease the activity of LPL, delaying TRL catabolism and thereby increasing remnant concentration [27,60,61,67]. Remnants are atherogenic and some become small-dense LDL; they all accumulate in the arterial intima, promoting atherosclerosis [24,87,137]. In humans, a minor additive effect of apoCIII is to precisely impair the uptake of remnants by the liver. Dietary intervention by reducing fructose consumption is a potent reducer of apoCIII, as we have recently shown [19].

### 7.2. Fructose and the Partition of TRL in the Fast-Fed Cycle

**Figure 9:**
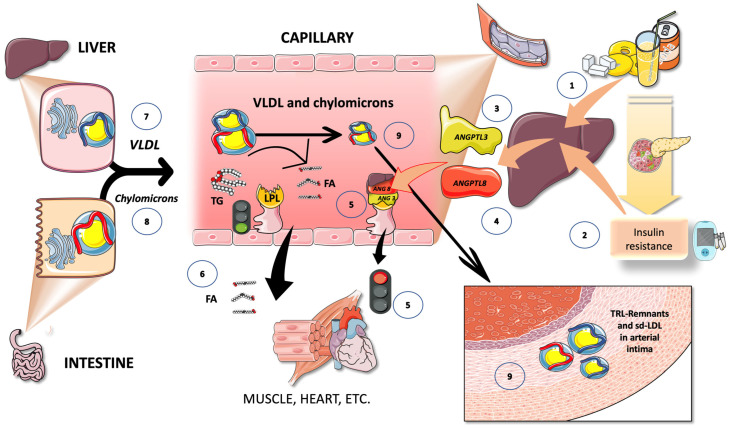
Fructose is associated with the increased expression of ANGPTL3, resulting in the impaired intravascular catabolism of VLDL and chylomicrons. Fructose, either directly (1) or indirectly, by producing insulin resistance (2) increases the liver’s expression of ANGPTL3 and -8 (3,4). These two molecules act in an endocrine fashion to inhibit LPL activity in peripheral tissues during the increased flux in the postprandial period (5), delaying the normal catabolism (6) of VLDL (7) and chylomicrons (8), and thereby increasing remnant concentration (9). Remnants are atherogenic and some become small-dense LDL; they all accumulate in the arterial intima, promoting atherosclerosis (9). This process has an additive effect to the effect on apoCIII, which is shown in Figure 5. This figure was partly generated using Servier Medical Art, provided by Servier, licensed under a Creative Commons Attribution 3.0 unported license.

In addition to the previously stated regulation of LPL activity, there is tighter control of LPL activity in various tissues, which facilitates the partition of a TRL load according to physiological demands. Chiefly, during fasting, lipids are preferentially taken up by oxidative tissues such as the cardiac and skeletal muscle, and storage in the adipocytes is not favored. Conversely, upon feeding, LPL activity in the adipocyte is much higher and, at the same time, its activity is reduced in the oxidative tissues. It is now obvious that LPL plays a crucial role in TG trafficking and partitioning. LPL activity increases in the white adipose tissue (WAT) after eating but decreases in the muscles; conversely, during fasting, LPL activity decreases in the WAT but increases in the muscles. However, a significant portion of the mechanism governing tissue-specific LPL activity throughout the feed-fast cycle is as yet unclear, although, as we shall see, considerable progress has been made in the past few years. LPL activity needs to be regulated at the tissue level to react to continually shifting metabolic conditions [138,139,140,141]. As significant tissue-specific regulators of lipolysis, ANGPTL3, ANGPTL4, and ANGPTL8 have been identified (Figure 9). In the postprandial period, ANGPTL3 (secreted all day long from the liver) acts in an endocrine way to inhibit lipoprotein lipase in the muscles and heart, an activity that is greatly enhanced during the postprandial period by ANGPTL8 (Figure 9) [80,138,139,141,142].

Recent research from our group has demonstrated that fructose restriction effectively lowers ANGPTL3, VLDL, and chylomicron remnants, as further discussed later [19,80].

## 8. Fructose and TRL in the Postprandial Period

Much of the research on lipoproteins, particularly in intervention studies (fructose research is not an exception), is conducted in the fasting state. However, we spend most of the day in the postprandial state, which is far more complicated to study, in part due to its complex kinetics, the difficulty in achieving the separation of VLDL vs. CM, and the involved and expensive experimental designs. Nevertheless, the past years have witnessed some progress in fructose research on the postprandial period [62,66,67,70,71,88,143,144,145].

### 8.1. Atherogenesis as a Postprandial Phenomenon

Almost half a century has passed since Zilversmit [143], after his seminal work on the matter, proposed that atherosclerosis is a postprandial phenomenon. Analytical challenges, however, have determined that most of our diagnostic and therapeutic targets only take into account fasting lipoprotein and lipid values. As we have seen, CM make up most of the wave of TRL that arise in the postprandial state. These TRL compete with hepatic TRL in the lipolytic and remnant elimination pathways, representing a dynamic “load” in addition to VLDL that is issued nearly continuously throughout the day.

The reason for postprandial increases in the blood levels of VLDL1 may be due to competition for the limited amount of LPL, for which large CM are the preferred substrate. This competition accounts for the strong correlation between fasting plasma TG levels and the degree of alimentary lipemia brought about by consuming fat [66,67]. A portion of the FA made from CM by LPL is also washed into the bloodstream as FA, rather than being absorbed into the tissues, as depicted in Figure 3. Spillover is the term for this process. It is now known that this postprandial input of dietary FA—roughly 5–30% of FA is added to the plasma FFA pool after a meal—is an important event. Since dietary FA are re-esterified after uptake by the liver, they may contribute to the postprandial rise in circulating VLDL [66,70]. Fructose, by directly and indirectly causing IR, is associated with boosted serum fluxes of FA [26].

### 8.2. Fructose Increases TRL Remnants: Their Role in “Residual Risk”

As stated above, recent studies have shown that fructose excess is associated with increases in the two major inhibitors of LPL, which leads to the accumulation of TRL remnants. Increased plasma TG levels may contribute significantly to the “residual risk” of ASCVD, according to recent genetic research. Since triglycerides per se are not known to contribute to atherogenesis, as we previously believed, a consensus has developed that the higher risk is caused, at least in part, by the greater quantity of cholesterol-enriched TRL remnant particles [59,146,147,148]. CM and large VLDL cannot penetrate the arterial wall due to their size; however, smaller CM and VLDL remnants can and do penetrate. In this sense, fructose excess may be detrimental because of its action on remnant buildup. One important factor raising the risk of ASCVD is the capacity of cholesterol-enriched TRL remnants to transfer their lipid cargo to macrophages, which are the cells in charge of cholesterol deposition in arterial plaques. Due to their pro-inflammatory effects, which are more severe per particle than those of modified LDL, as well as their unregulated uptake by macrophage scavenger receptors, remnant lipoproteins that have undergone oxidative alteration play a substantial role in atherogenesis [23,87,137]. Among other atherogenic traits, remnant lipoproteins also carry thrombogenic substances, activate monocytes, and promote endothelial cell death and smooth muscle cell proliferation.

Our recent studies have shown that isocaloric fructose restriction reduced the AUC of TG, apoCIII, ANGPTL3, and apoB48, showing a continuous benefit throughout most of the postprandial period [19]. This has been corroborated by another study, which goes on to suggest that the fructose-induced delayed catabolism of TRL is even more relevant than increased production [40,113].

### 8.3. LPL Inhibitors Are the Targets of New Approaches to Residual Risk

The remarkable importance of these processes is underscored by the trend in lipid-lowering medications over the past five years, where attention has been placed on developing substances that boost TRL lipolysis. Due to their recognized functions in controlling LPL activity and the availability of genetic data supporting the proof of concept that lowering the concentration of these targets is expected to result in decreased ASCVD risk, ApoCIII and ANGPTL3 are currently the primary targets [56,57,135,138,142,147,148,149,150]. The two most promising strategies are RNA silencing technology and monoclonal antibody inhibition. Phase II and phase III clinical trials are currently testing antisense oligonucleotides that target APOC3 mRNA (volanesoren and olezarsen) and ANGPTL3 mRNA (vupanorsen) or Evinacumab, a monoclonal antibody that targets ANGPTL3. In both animal and human investigations, these substances lower the plasma levels of apoC-III and ANGPTL3 by about 70% and 80%, respectively. Evanicumab is used to treat homozygous familial hypercholesterolemia in patients.

Interestingly, we and others have shown that short-duration fructose restriction reduces apoCIII and ANGPTL3 by at least 30%, showing the significance of this simple lifestyle change in achieving manifest metabolic benefits [19].

## 9. Isocaloric Fructose Restriction Reverts Most of the Lipid Disturbances in Adolescents

**Figure 10:**
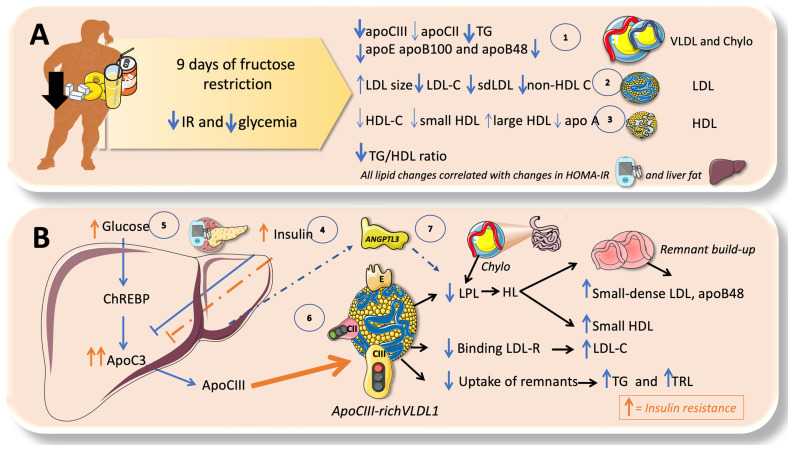
Nine days of isocaloric fructose restriction revert dyslipidemia in our studies. (**A**). Fructose restriction to the levels recommended by the AHA in adolescents led to changes in VLDL and CM (1), LDL (2), and HDL (3), as depicted in the Figure, all changes that are associated with changes in IR, liver fat, and DNL. (**B**). The main proposed mechanisms (other than excess production) explain our results, which agree with previous and posterior results by others in overfeeding studies. The role of IR (4) and hyperglycemia (5) in apoCIII-enriched large VLDL1 production is illustrated (6). ANGPTL3 is also increased (7) and both contribute to delay catabolism, which explains the findings. This figure was partly generated using Servier Medical Art, provided by Servier, licensed under a Creative Commons Attribution 3.0 unported license.

Before recapitulating the synergic impact of fructose (sugar) excess on both TRL production and catabolism, let us illustrate the reversibility of both pathways via fructose restriction. We have conducted a 9-day fructose restriction study (reducing fructose consumption in obese adolescents to AHA levels). Care was exerted so that the study would be isocaloric, meaning that calories are out of the picture. The study explored lipids, liver fat, insulin kinetics, and DNL with cutting-edge technology, and both the fasting and postprandial periods were explored. Figure 10 summarizes the lipoprotein results [19,21,22,30,44]. Adolescents who restricted their fructose intake to the amounts advised by the AHA experienced changes in their VLDL and also CM, LDL, and HDL, all of which were correlated with changes in their IR, liver fat, and DNL. One of the main hypothesized mechanisms (other than excessive production) to account for our findings—which are consistent with earlier and later findings by other researchers in overfeeding investigations—is fructose-induced hyperinsulinemia, where hyperglycemia and IR affect the formation of apoCIII-enriched large VLDL1. The effects are compounded by the elevated levels of ANGPTL3, further compromising delayed catabolism. The reductions in DNL and liver fat found in the study show the effect on the upstream component of TG production. Our findings are consistent with a recent meta-analysis comparing high-sugar to low-sugar diets, which showed that excessive free sugar consumption raises TG, cholesterol, and LDL-C without increasing body weight. Recent research shows that when consumed together as HFCS, glucose and fructose interact. This may offer crucial mechanistic information about the pathophysiology of excessive sugar consumption [19,21,22,30,31,44].

Further studies in other populations and of a longer duration, along with further analysis of the ultimate mechanism for insulin resistance and postprandial interactions of TRL with LPL, are warranted.

## 10. High Sugar Diets Promote TRL Accumulation and Increase in Atherogenic Remnants by Interfering with Both Their Production and Catabolism: Recapitulation

**Figure 11:**
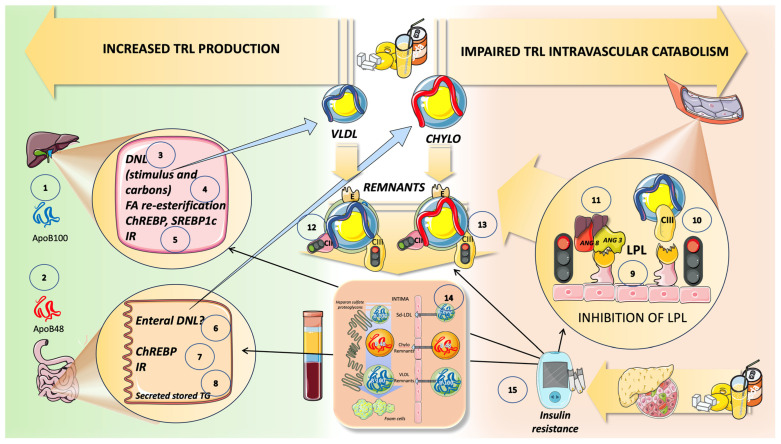
The sugar double-hit on lipoproteins: increased production and decreased catabolism. Sugar overfeeding (as seen in the Western diet) leads to the enhanced lipogenic effect of fructose being “optimized” by glucose-concomitant availability and insulin resistance. To the left of the figure, we summarize the processes discussed in this review that lead to both liver (1) and intestine (2) overproduction of triglyceride-rich lipoproteins. In the liver, fructose (especially in the presence of glucose) promotes DNL (3) either by providing the carbons for fatty acids (4) or by producing excess glycerol backbone for TG, or both. Fructose-1-P, a key specific fructose metabolite, integrates glucose and fructose metabolism, as do ChREBP and SREBP1c (5) via insulin resistance. Although the evidence is less robust, DNL occurs in the enterocyte in humans (6) when under similar stimulation by ChREBP and IR (7). Additionally, stored enterocyte TG are produced when the sweet taste receptors are stimulated (8). To complicate matters, recent research demonstrates that excessive sugar ingestion causes LPL to be inhibited (6), which results in delayed TRL catabolism, as shown on the right of the Figure (9–11). Studies on fructose overfeeding and restriction in humans show that fructose raises the levels of two key LPL inhibitors: apoCIII (10), which is active throughout the day, and ANGPTL3/8 (11), which predominates in the postprandial period. The build-up of apoB100 and apoB48 residues shows how the two processes work together in (12) and (13). The importance of remnant accumulation in the arterial intima is highlighted in (14) and the effect of IR at multiple points is illustrated in (15). This figure was partly generated using Servier Medical Art, provided by Servier, licensed under a Creative Commons Attribution 3.0 unported license.

The increased lipogenic action of fructose caused by excessive sugar consumption (as found in the Western diet) is “optimized” by the concurrent availability of glucose and insulin resistance. The mechanisms covered in this review that result in the overproduction of triglyceride-rich lipoproteins in the liver and gut are summarized to the left of Figure 11. Fructose stimulates DNL in the liver, either by supplying the carbons for fatty acids, by overproducing the glycerol backbone for TG, or by doing both. Through insulin resistance, ChREBP and SREBP1c, as well as fructose-1-P, a crucial fructose metabolite, integrate glucose and fructose metabolism. Additionally, stored enterocyte TG are produced when the sweet taste receptors are stimulated. To complicate matters, recent research demonstrates that excessive sugar ingestion causes LPL to be inhibited, which results in delayed TRL catabolism, as shown on the right of Figure 11. Studies on fructose overfeeding and restriction in humans show that fructose raises the levels of two key LPL inhibitors: apoCIII, which is active throughout the day, and ANGPTL3/8, which predominates in the postprandial period. The build-up of apoB100 and apoB48 residues shows how the two aforementioned processes work together. A consensus has emerged that a higher risk is brought on, at least in part, by the higher number of cholesterol-enriched TRL remnant particles, since triglycerides per se are not known to contribute to atherogenesis as we previously stated. Due to their size, CM and large VLDL cannot pass through the artery wall; however, smaller CM and VLDL remnants can and do pass through, as we illustrate in Figure 11.

Over half a century ago, it was suggested that the diabetes epidemic that we are currently experiencing may be caused by mutations picked up during historical periods of hunger. We now know that some of them are related to fructose metabolism [151]. Indeed, there is another, less widely explored source of fructose, namely, the endogenous pathway or the polyol pathway. Aldose reductase reduces glucose into sorbitol, which sorbitol dehydrogenase then breaks down into fructose. Additionally, high salt diets (which increase osmolality) can stimulate aldose reductase. Other stimuli for aldose reductase include heat, tissue hypoxia, oxidative stress, fructose, and uric acid [151,152]. The current literature supports the notion that mutations affecting the endogenous fructose pathway may have improved the ability of early humans to survive in environments with meager food supplies (by stimulating lipid synthesis, water retention, and regulating blood pressure), but they also increase our risk of obesity, dyslipidemia, diabetes, CVD, and hypertension in modern times. As we have shown, the introduction of refined sugar and HFCS has significantly increased fructose consumption. Furthermore, the growing linkage of fructose as a fuel for different malignancies is probably explained by its capacity to activate aerobic glycolysis (the Warburg effect) [151]. More research is needed to assess the quantitative role of the polyol pathway vis-à-vis the current tsunami of dietary fructose.

## 11. Conclusions

In this review, we concentrated on the role of fructose in the development of cardiometabolic dyslipidemia (an increase in serum triglyceride-rich lipoproteins (TRL): VLDL, chylomicrons, and their remnants), bringing together the most recent human data that demonstrates the critical interaction between glucose and fructose to increase synthesis while lowering the catabolism of these particles in a synergistic downward spiral. We evaluated fructose metabolism in the liver and intestines, as well as the consequences of dysregulated fructolysis and the glucose-induced activation of ChREBP. Because it acts as a signaling molecule to boost the production of fat, fructose-1-P, the first metabolite of fructose catabolism, is highlighted, together with the ensuing crosstalk with glucose metabolism. We underline the importance of this fructose/glucose relationship to the liver, which boosts the generation of VLDL, triglycerides, and de novo lipogenesis. Conversely, we draw attention to recent studies showing how fructose influences lipoprotein lipase activity by raising the concentration of inhibitors such as apoCIII and ANGPTL3, which decrease the catabolism of VLDL and CM, leading to the buildup of their atherogenic remnants. In the end, atherogenesis is promoted by a dual, synergistic, and detrimental activity. We highlight that a major dietary contributor to dyslipidemia, which, in turn, affects the risk of cardiovascular disease (CVD), is the continuous, daily, and chronic transit of associated fructose and glucose (as sugar or HFCS) to the liver. We provide recent mechanistic evidence that strongly supports public health organizations’ activities targeted at limiting sugar intake, including the introduction of dietary guidelines addressing “safe” limits for sugar consumption, given the growing concerns regarding the link between sugar consumption and cardiometabolic disease.

## Data Availability

Not applicable.
